# Rats sniff out pulmonary tuberculosis from sputum: a diagnostic accuracy meta-analysis

**DOI:** 10.1038/s41598-021-81086-x

**Published:** 2021-01-21

**Authors:** Reem Kanaan, Nelli Farkas, Péter Hegyi, Alexandra Soós, Dávid Hegyi, Katalin Németh, Orsolya Horváth, Judit Tenk, Alexandra Mikó, Andrea Szentesi, Márta Balaskó, Zsolt Szakács, Andrea Vasas, Dezső Csupor, Zoltán Gyöngyi

**Affiliations:** 1 Department of Public Health Medicine, Medical School, University of Pécs, Szigeti út, 12, 7624 Pécs, Hungary; 2 Institute of Bioanalysis, Medical School, University of Pécs, Pécs, Hungary; 3 Institute for Translational Medicine, Medical School, University of Pécs, Pécs, Hungary; 4 First Department of Medicine, Medical School, University of Pécs, Pécs, Hungary; 5 János Szentágothai Research Centre, University of Pécs, Pécs, Hungary; 6 Momentum Gastroenterology Multidisciplinary Research Group, Hungarian Academy of Sciences, University of Szeged, Szeged, Hungary; 7 Doctoral School of Clinical Medicine, University of Szeged, Szeged, Hungary; 8 Department of Pharmacognosy, Faculty of Pharmacy, University of Szeged, Szeged, Hungary; 9 Interdisciplinary Centre of Natural Products, University of Szeged, Szeged, Hungary

**Keywords:** Microbiology, Diseases

## Abstract

In Sub-Saharan Africa, African giant pouched rats (*Cricetomys gambianus*) are trained to identify TB patients by smelling sputum. We conducted a systematic review and meta-analysis of the data to see if this novel method is comparable to traditional laboratory screening and detection methods like Ziehl–Neelsen stain-based assays (ZN) and bacterial culture. The search and data processing strategy is registered at PROSPERO (CRD42019123629). Medline via PubMed, EMBASE, Web of Science, and Cochrane Library databases were systematically searched for the keywords “pouched rat” and “tuberculosis”. Data from 53,181 samples obtained from 24,600 patients were extracted from seven studies. Using sample-wise detection, the sensitivity of the studies was 86.7% [95% CI 80.4–91.2%], while the specificity was 88.4% [95% CI 79.7–93.7%]. For patient-wise detection, the sensitivity was 81.3% [95% CI 64.0–91.4%], while the specificity was 73.4% [95% CI 62.8–81.9%]. Good and excellent classification was assessed by hierarchical summary receiver-operating characteristic analysis for patient-wise and sample-wise detections, respectively. Our study is the first systematic review and meta-analysis of the above relatively inexpensive and rapid screening method. The results indicate that African giant pouched rats can discriminate healthy controls from TB individuals by sniffing sputum with even a higher accuracy than a single ZN screening.

## Introduction

Tuberculosis (TB) is the world’s leading cause of death by an infectious disease despite the 90-year and 60-year availability of vaccine and drug therapy, respectively^1^. World Health Organization (WHO), published an estimated 10.0 million (range 9.0–11.1 million) new cases of TB, an estimated 1.2 million (range 1.1–1.3 million) TB deaths among HIV-negative people, and a further 251 000 deaths (range 223,000–281,000) among HIV-positive people in 2018^2^.

*Mycobacterium tuberculosis* is the main causative agent of TB and primarily infects the lungs^2^. However, extrapulmonary TB, which occurs by lymphatic or blood spread of *Mycobacteria* at the time of primary infection, can affect other organs such as the pleura, lymph nodes, genitourinary tract, abdomen, skin, bones, joints, and meninges^3–6^. Following exposure to *M. tuberculosis*, most individuals remain disease-free but may carry a latent TB infection (LTBI), the main reservoir for tuberculosis reactivation. A minority of LTBI individuals [5–15%] will progress to active TB^7^. TB progression is enhanced dramatically for people co-infected with HIV^8^. United States guidelines recommend screening for LTBI in all HIV-infected patients^9^. Patients with immune diseases and patients receiving biological therapy also have enhanced TB progression compared to the general population^10^.

Treatment of TB requires multiple drugs for several months. The extended drug regimens are demanding on the health care systems, particularly in low- and middle-income countries, where the disease burden often far surpasses local resources^1^.

Developing a fast and affordable tool with reasonable sensitivity and specificity for TB screening, prognosis, and detection of drug resistance is a challenge^11^. Currently, several laboratory-based methods exist including the Ziehl–Neelsen stain-based, direct microscopy (ZN), the Xpert MTB/RIF assay, the lipoarabinomannan antigen test, nucleic acid amplification tests, mass spectroscopy, surface-enhanced Raman spectroscopy, detection of TB-specific volatile organic compounds (VOCs) or metabolites, microfluidics, and electrochemical approaches^12^. Each of these methods has merits and shortcomings. No test provides all the information required for diagnosis and treatment monitoring^13^.

In Sub-Saharan Africa, the continent with a high TB-burden, researchers are applying a new TB detection tool using African giant pouched rats (*Cricetomys gambianus*) to identify TB patients. By smelling patient sputum, they presumably recognise TB-specific VOCs profile. VOCs are organic chemicals with a high vapour pressure at room temperature, resulting in the evaporation or sublimation of molecules into the air^14^. Inhalation of these molecules stimulates the olfactory receptor neurons to transmit signals to the olfactory cortex of the brain^14^. Odour perception results in task-dependent sniffing patterns in the context of odour-guided behaviour^15^. This method seems to be cost-effective and reliable. To our knowledge, there is no meta-analysis of the method published to date; therefore, we present a systematic review and meta-analysis regarding the sensitivity and specificity of using African giant pouched rats as an additional tool to detect pulmonary TB.

## Material and methods

### Search strategy and selection process

Our systematic review and meta-analysis followed the protocol registered at PROSPERO (CRD42019123629). Criteria from “Interpreting results and drawing conclusions. Cochrane Handbook for Systematic Reviews of Diagnostic Test Accuracy Version 0.9” and the Preferred Reporting Items for Systematic Reviews and Meta-Analyses (PRISMA-P) protocols were considered^16,17^. PRISMA checklist is enclosed (Supplementary Table S1).

Medline via PubMed (www.ncbi.nlm.nih.gov/pubmed), EMBASE (www.embase.com), Web of Science Core Databases (Web of Science, Thomson Reuters; www.webofknowledge.com), and Cochrane Central Register of Controlled Trials (Cochrane Library, Wiley; www.cochranelibrary.com) databases were searched without restrictions. The keywords used for the searches were “tuberculosis” and “pouched rats.” The database search was concluded on 5 September 2019. The search results were exported to EndNote. The inclusion criteria were based on the method of validation and the type of sampling (sample-wise or patient-wise), and the presence or calculability of data for sensitivity and specificity. Sample-wise data identified samples that were positive or negative, while patient-wise data identified patients with or without TB. Studies that failed to mention the exact sample size or did not report quantitative data were excluded.

Two reviewers independently retrieved information from the databases and screened references of relevant research articles, reviews, and additional data sources for relevant publications. A third reviewer was consulted if required for a consensus. After removing duplicate records from the search results, candidates were selected by reading the titles, abstracts or full texts of the retrieved records. Potential articles were analysed, and non-relevant studies were excluded. Authors, year of publication, sample size, significance, country of study, and other available data about the study design and participants were registered for the meta-analysis.

### Quality of included studies

Assessment of methodological quality (risk of bias) was performed by two authors based on the Mays and Pope model^18^. A third reviewer was consulted if required for a consensus. The included studies involved answering questions concerning the clarity of research, blindness, representative and adequate sampling, control of confounding variables, research design suitable to answer the research question, ethical clearance, reporting of overall sensitivity percentage, reporting of overall specificity percentage, and reporting of limitations.

### Data analysis

Statistical analysis was performed with Stata v15.1 software using the MIDAS and METANDI modules. In the present meta-analysis, we generated 2 × 2 tables from the data of selected papers (Supplementary Table S2). Patient-wise and sample-wise subgroups were analysed. Heterogeneity was assessed using the I^2^ measure and the corresponding χ^2^ test, with p < 0.1 indicating significant heterogeneity. I^2^ values of 25%, 50%, and 75% were estimated as low, moderate, and high, respectively. First, we collected sensitivity and specificity data from the studies. Second, we either calculated or extracted true-positive values, false-positive values, false-negative values, and true-negative values (Supplementary Table S3). Third, by plotting the true-positive rate (TPR) (sensitivity) against the false-positive rate (FPR) (1 − specificity) at various threshold settings, hierarchical summary ROC curve was created, using the two-level mixed logistic regression modell^19^. Estimation of sensitivity and specificity points on the curve was done at the 95% confidence interval. The diagnostic odds ratio (DOR) was computed at a 95% confidence interval as well. According to the AUC (area under the curve) value, the test was classified as follows: 0.5–0.6 failed, 0.6–0.7 poor, 0.7–0.8 fair, 0.8–0.9 good, and > 0.9 excellent^20^. Deeks’ Funnel Plot Asymmetry test was performed to reveal possible publication bias.

## Results

### Identification of eligible studies

**Figure 1:**
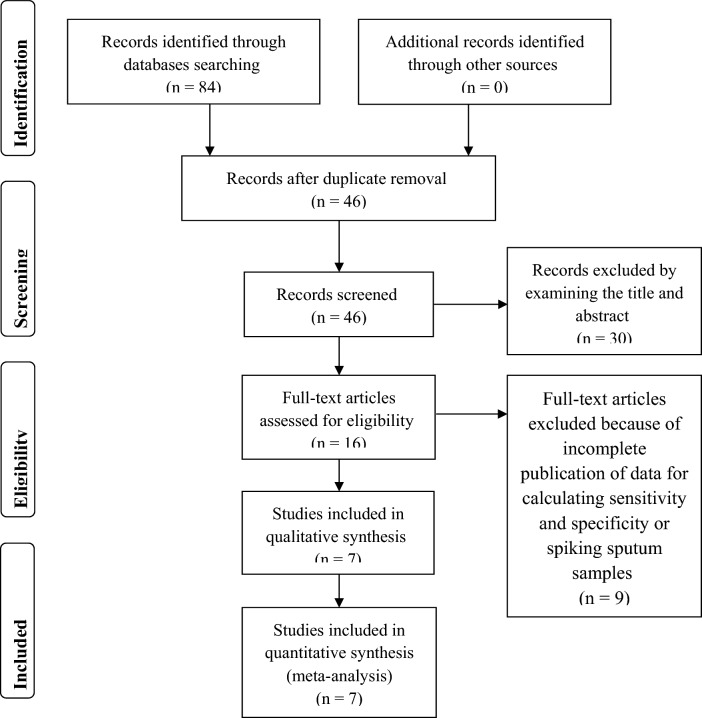
Study selection. PRISMA 2019 flow diagram. Database searches identified 84 records. Of the overall obtained studies, 38 were duplicates. From the 46 remaining studies, 30 were excluded either by examining the title or by examining the abstract. Of the remaining 16 studies, full-text examination resulted in identifying seven publications that are eligible for meta-analysis inclusion criteria.

For our systematic review and meta-analysis, we examined Medline via PubMed, EMBASE, Web of Science, and Cochrane Library databases resulting in 84 records: 20, 25, 39, and 0 publications, respectively. Of the 84 publications, the removal of duplicates resulted in 46 publications from which another 22 publications were excluded by examining the title, and an additional eight were excluded by examining the abstract. From the remaining 16 studies, seven publications^21–27^ met the meta-analysis inclusion criteria as shown using the PRISMA method (Fig. 1).

### Characteristics of included studies

A total of 24,600 human subjects and 53,181 samples were screened between the seven studies (Table 1). All seven studies collected sputum samples in Direct Observation Treatment Short Course (DOTS) centres in Dar el Salaam, Tanzania. Technicians at DOTS centres prepared microscopic slides from sputum samples. After stained using the ZN method, the samples were evaluated by light microscopy. Remaining sputum was frozen and sent to Anti-Persoonsmijnen Ontmijnende Product Ontwikkeling (APOPO) for rat assessment^27^. Trained African pouched rats sniffed heat-inactivated sputum samples. They showed positive samples by holding their head at the well of sample container. A second ZN microscopic analysis at APOPO re-examined any sample reported as TB-negative by a DOTS centre but TB–positive by two or more rats^27^. The accuracy of detection by rats was calculated after the second ZN analysis.

**Table 1:** Samples, subjects, and methods used for comparison with the rat-sniffing method.

First author	Year	Country	Study design	Sample type	Standard method	Number of subjects	Number of samples
Mahoney AM,	2011	Tanzania	second-line screening	Patient-wise, Sample-wise	Ziehl–Neelsen, FM microscopy	12,329	26,665
Mgode GF,	2012	Tanzania	second-line screening	Patient-wise	Ziehl–Neelsen, culture; mixed	284	
Reither K,	2015	Tanzania	prospective cohort	Patient-wise	Ziehl–Neelsen, culture	246	
Weetjens BJ,	2009	Tanzania	second-line screening	Sample-wise	Ziehl–Neelsen, culture		2,597
Poling, A	2010	Tanzania	second-line screening	Patient-wise, sample-wise	Ziehl–Neelsen	10,523	23,101
Mulder C,	2017	Tanzania	paired accuracy study	Patient-wise	culture, Xpert, LED FM	771	
Edwards TL,	2016	Mozambique	second-line screening	Patient-wise, sample-wise	Ziehl–Neelsen, LED FM	447	818

LED FM, Light-emitting diode fluorescence microscopy; Xpert, MTB/RIF test that detects *Mycobacterium tuberculosis* complex (MTBC) and resistance to rifampicin (RIF) on the GeneXpert multi-disease platform.

### Analysis of results

**Figure 2:**
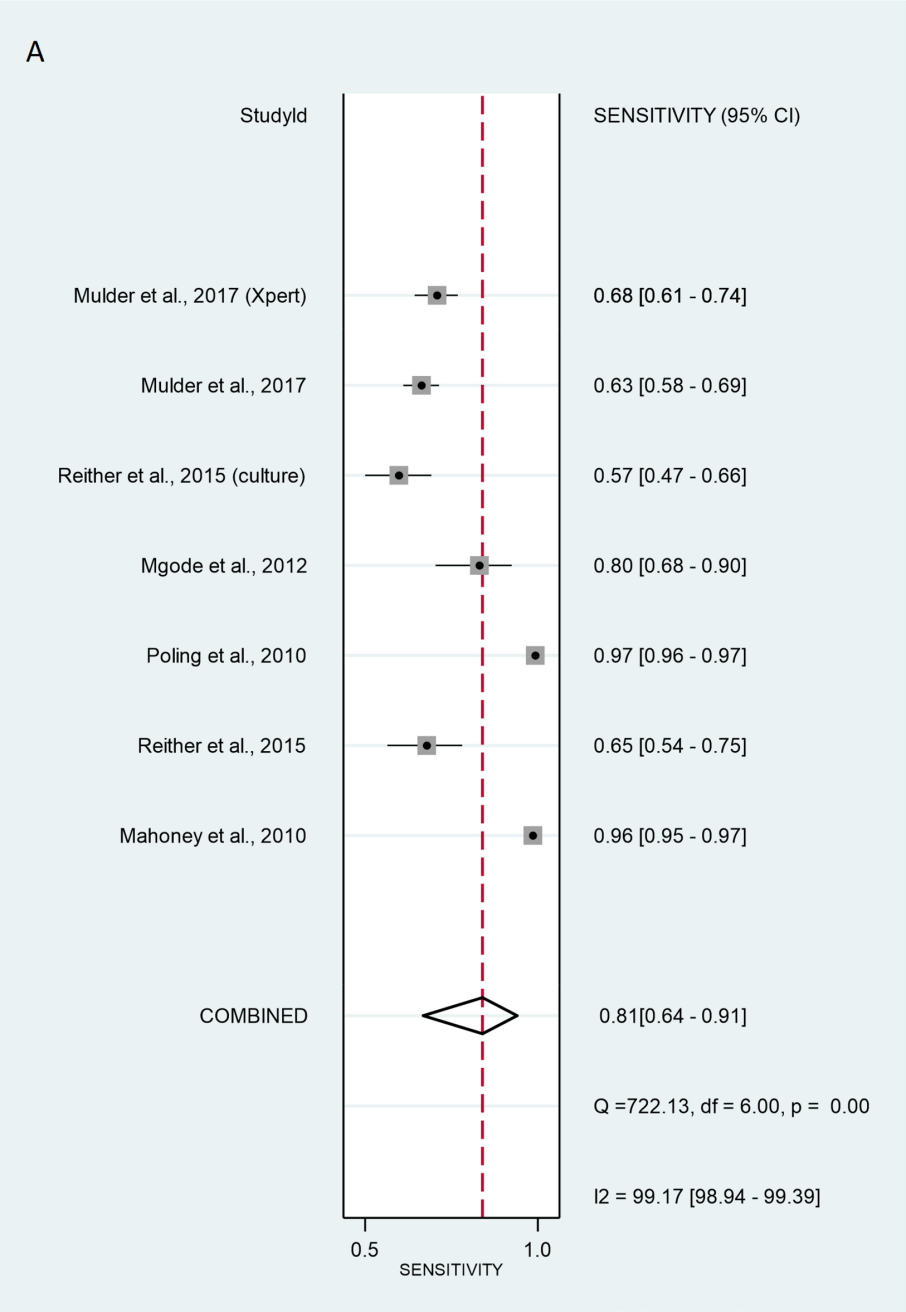

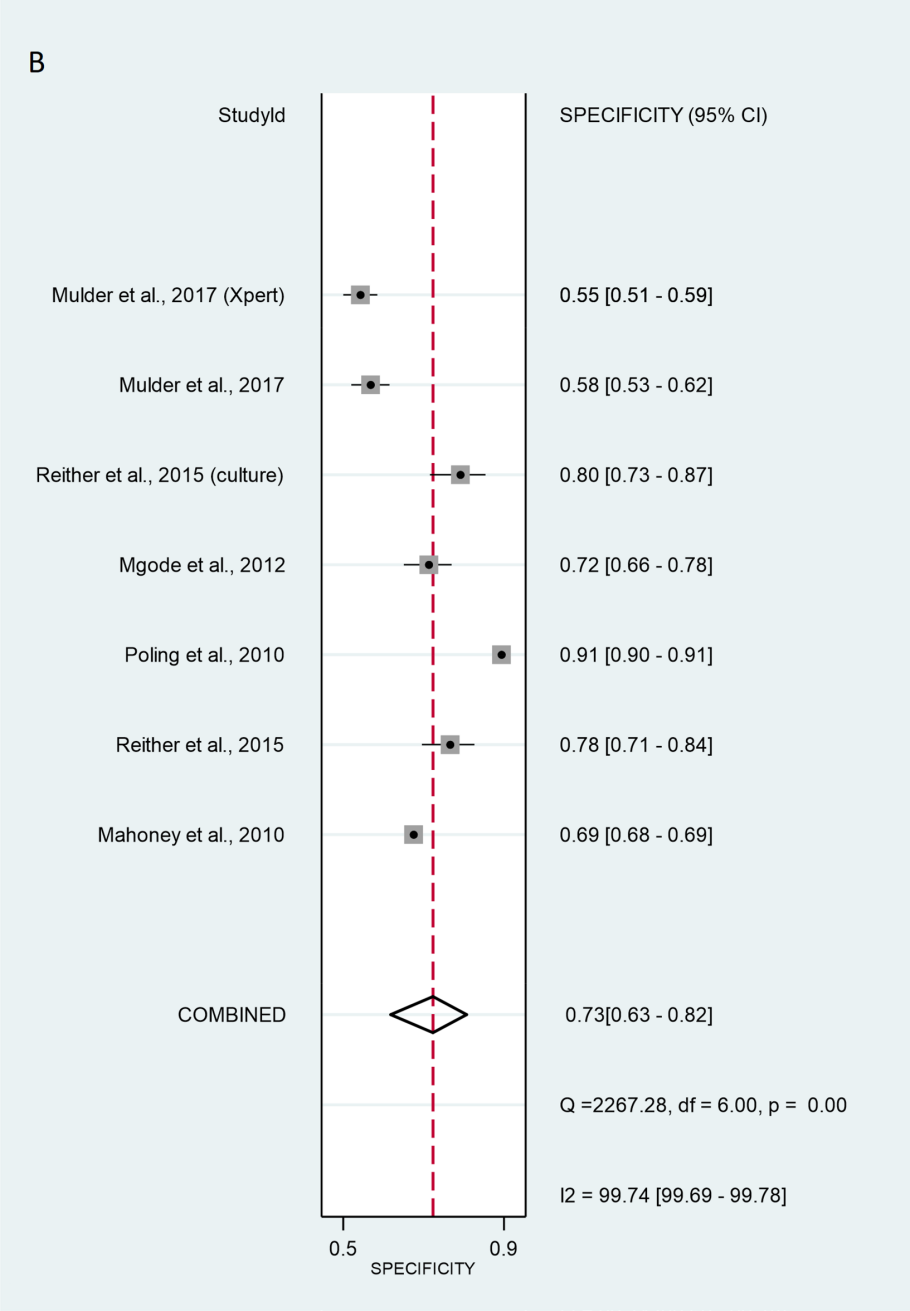
Sensitivity (**A**) and specificity (**B**) of individual studies are displayed by squares. The overall values are displayed by rhombus. Error bars indicate confidence interval of 95% [95% CI]. The reference method was Ziehl–Neelsen stain-based, direct microscopy, except for other method is indicated in brackets.

**Figure 3:**
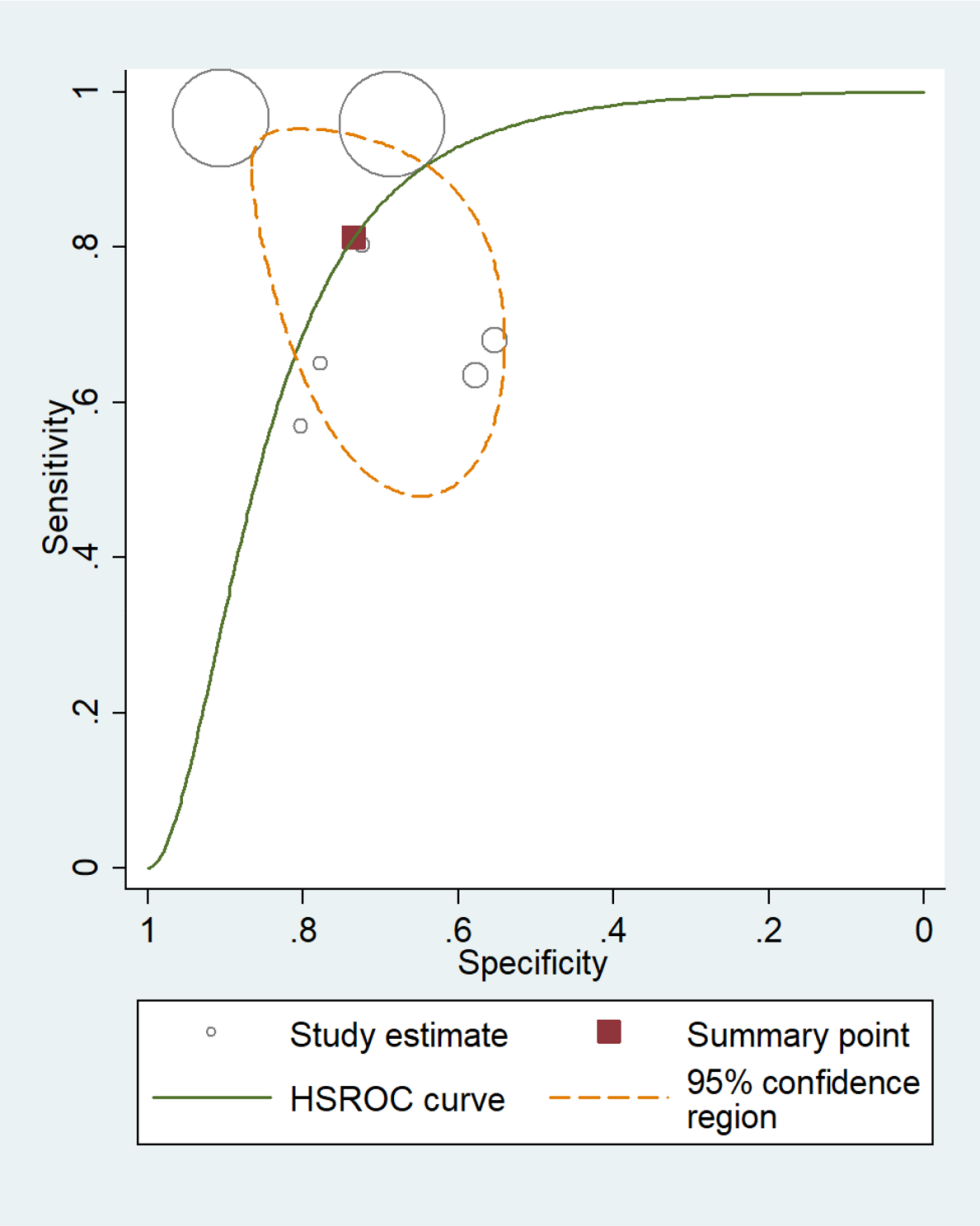
The size of circles indicates the number of patients in a single study. The dark red square shows the sensitivity and specificity summary. The dashed line indicates the 95% confidence region. A meta-analysis of all seven studies revealed that the summary of sensitivity was 81.3% [95% CI 64.0–91.4%] and the specificity was 73.4% [95% CI 62.8–81.9%]. The diagnostic odds ratio was 12.0 [95% CI 3.58–39.9]. According to the AUC 0.82 [95% CI: 0.79–0.86] value, the test was classified as good. HSROC, hierarchical summary receiver-operating characteristic; AUC, area under the curve.

The validation method for the six studies with patient-wise data was as follows: three of studies used the ZN method (Mahoney^26^, Reither^23^ and Poling^27^. Mgode et al.^20^ used the ZN method and cultures. Two studies, Reither ^23^ and Mulder ^29^, used only cultures. Mulder et al.^29^ additionally used Xpert MTB/RIF. The SROC curve for the diagnostic performance of rats compared to ZN alone, ZN and culture, culture alone, and Xpert MTB/RIF revealed that the sensitivity of patient-wise analysis was 81.3% [95% CI 64.0–91.4%] and the specificity was 73.4% [95% CI 62.8–81.9%] (Fig. 2). Analysing positive (true positive) and negative (true negative) likelihood ratios, the LR + and LR − were 3.05 [95% CI 1.92–4.86] and 0.10 [95% CI 0.06–0.17], respectively. The DOR equalled 12.0 [95% CI 3.58–39.9] (Fig. 3), indicating statistical significance. Thus, rats discriminated properly between sputa of healthy and TB-infected individuals. However, significant heterogeneity was calculated at sensitivity and specificity with values of I^2^ = 99.17% [95% CI 98.94–99.39%], p < 0.001, and I^2^ = 99.74% [95% CI 99.69–99.78%], p < 0.001 respectively.

**Figure 4:**
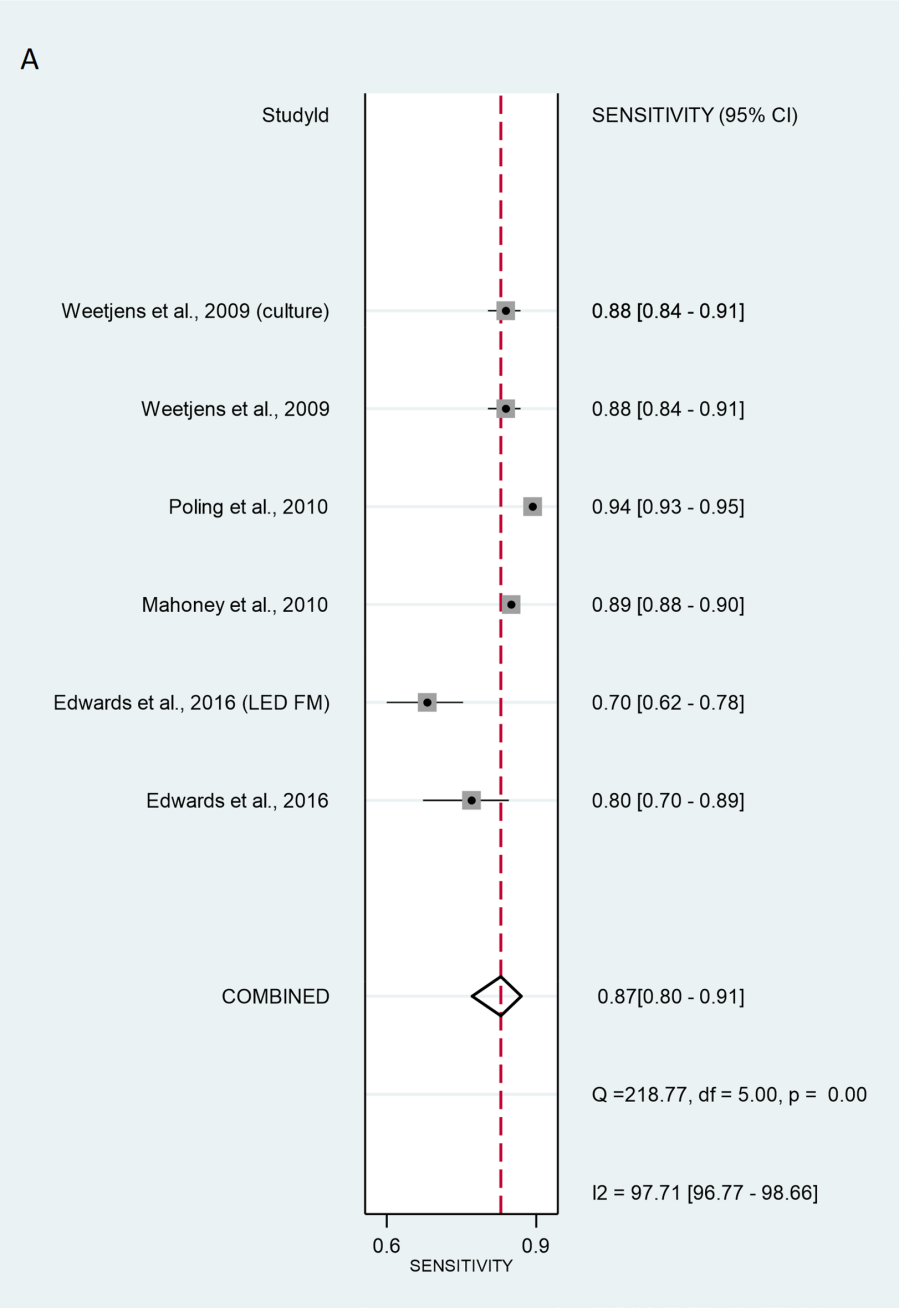

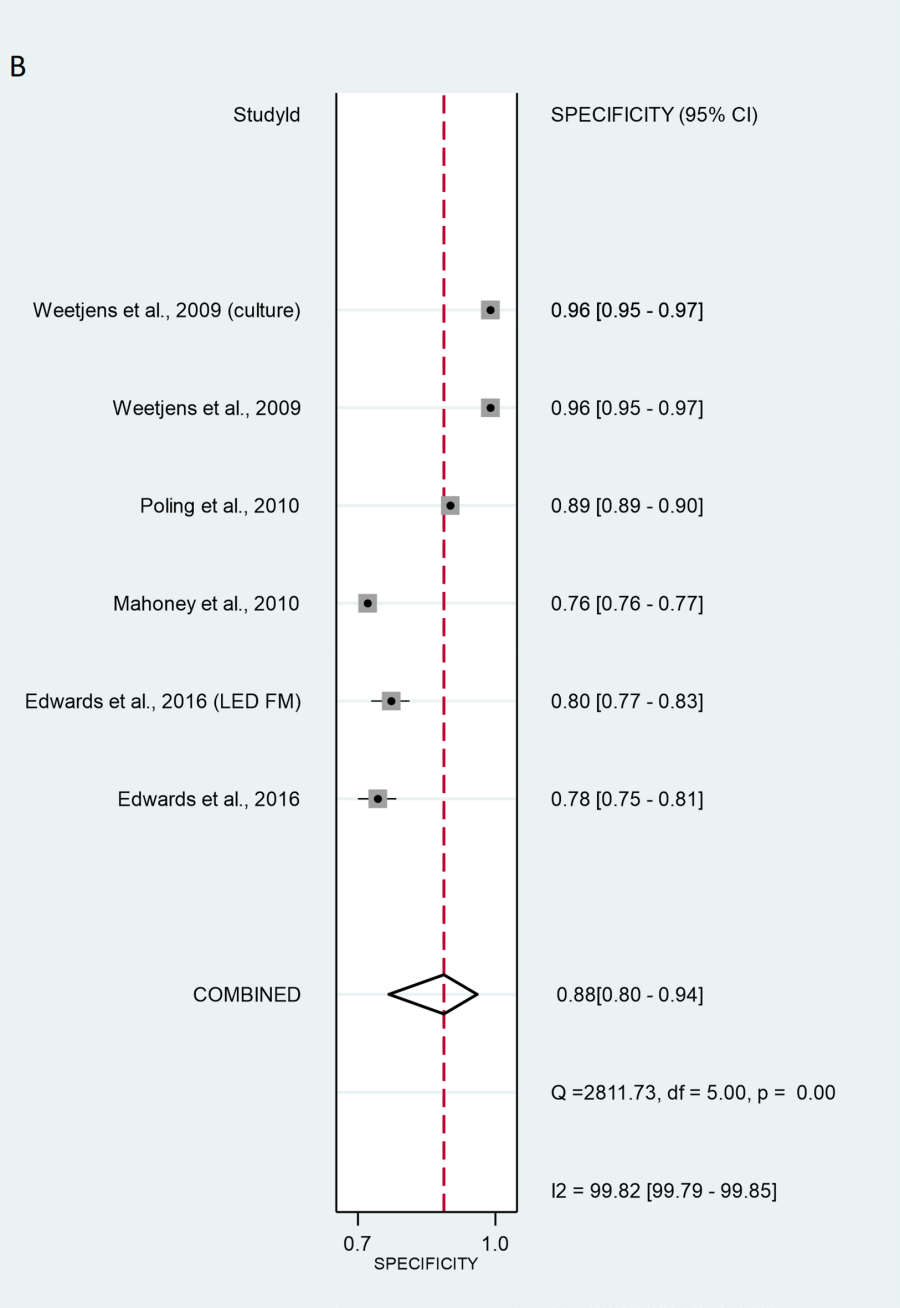
Sensitivity (**A**) and specificity (**B**) of individual studies are displayed by squares. The overall values are displayed by rhombus. Error bars indicate confidence interval of 95% [95% CI]. The reference method was Ziehl–Neelsen stain-based, direct microscopy, except for other method is indicated in brackets.

**Figure 5:**
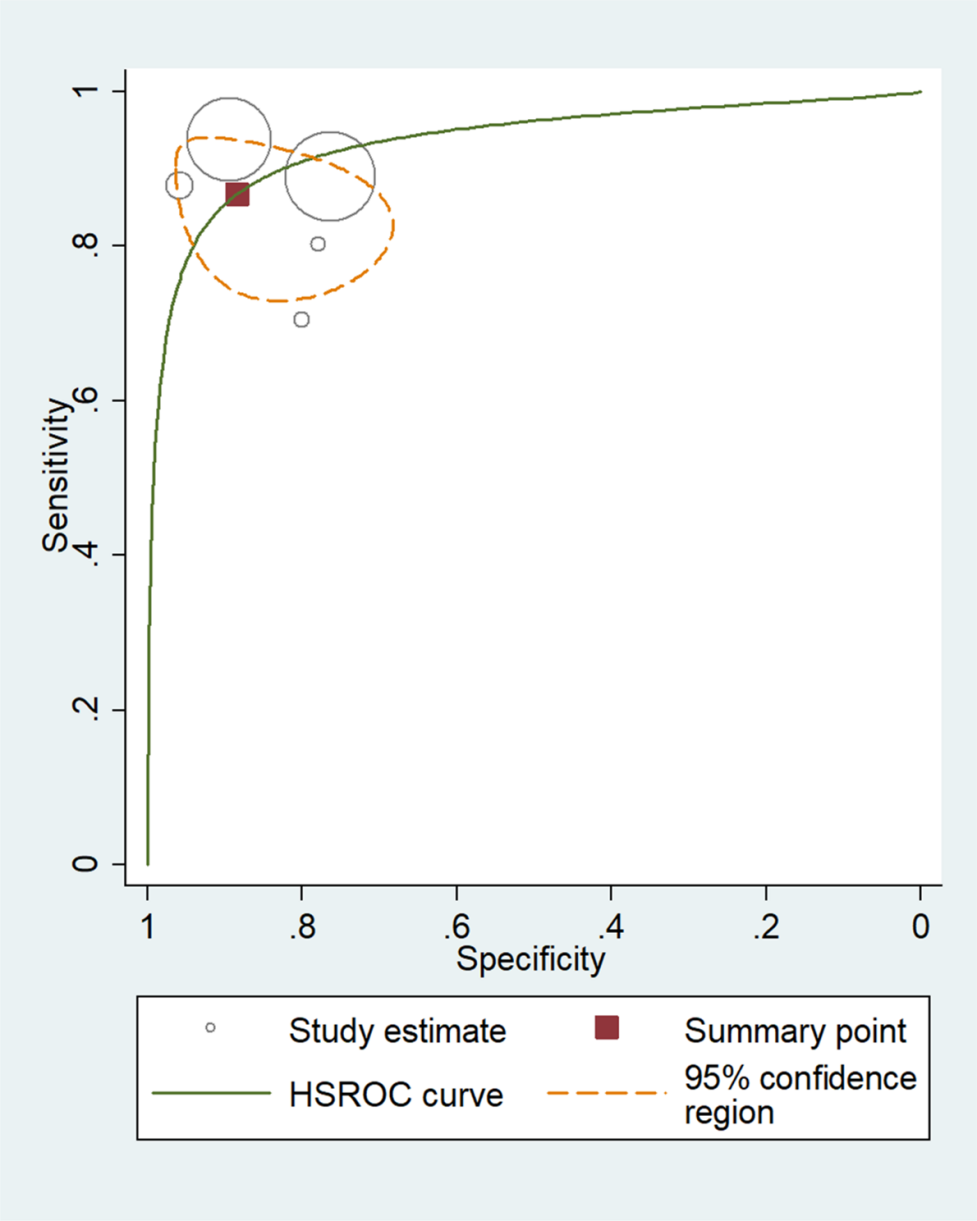
Diagnostic performance of screening using rats compared to ZN microscopic analysis, LED FM, and culture. The size of circles indicates the number of patients in a single study. The dark red square shows the sensitivity and specificity summary. The dashed line indicates the 95% confidence region. Meta-analysis summary of the six studies (five circles are seen, because two studies are exactly overlapping) shows that the sensitivity of rat screening was 86.7% [95% CI 80.4–91.2%] and specificity was 88.4% [95% CI 79.7–93.7%]. The summary for positive LR + and LR − was 7.47 [95% CI 4.05–13.8] and 0.15 [95% CI 0.1–0.23], respectively. The diagnostic odds ratio was 49.8 [95% CI 19.5–127]. According to the AUC 0.93 [95% CI: 0.91–0.95] value the test was classified as excellent. HSROC, hierarchical summary receiver-operating characteristic; AUC, area under the curve.

For sample-wise data, the method of validation used by Mahoney^26^, Weetjens^21^, Poling^27^, and Edwards^25^ was ZN. Edwards^25^ used LED FM, while Weetjens^21^ used cultures. Together, the six sample-wise studies revealed rat screening sensitivity of 86.7% [95% CI 80.4–91.2%] and specificity of 88.4% [95% CI 79.7–93.7%]. Total LR + and LR– were 7.47 [95% CI 4.05–13.8] and 0.15 [95% CI 0.1–0.23], respectively (Fig. 4). DOR was 49.8 [95% CI 19.5–127], indicating that the test discriminated properly between positive and negative samples (Fig. 5). Values of sensitivity and specificitiy were I^2^ = 97.71% [95% CI 96.77–98.66%], p < 0.001 and I^2^ = 99.82% [95% CI 99.79–99.85%], p < 0.001 respectively. These data indicate a considerable heterogeneity.

### Assessment of risk of publication bias

**Figure 6:**
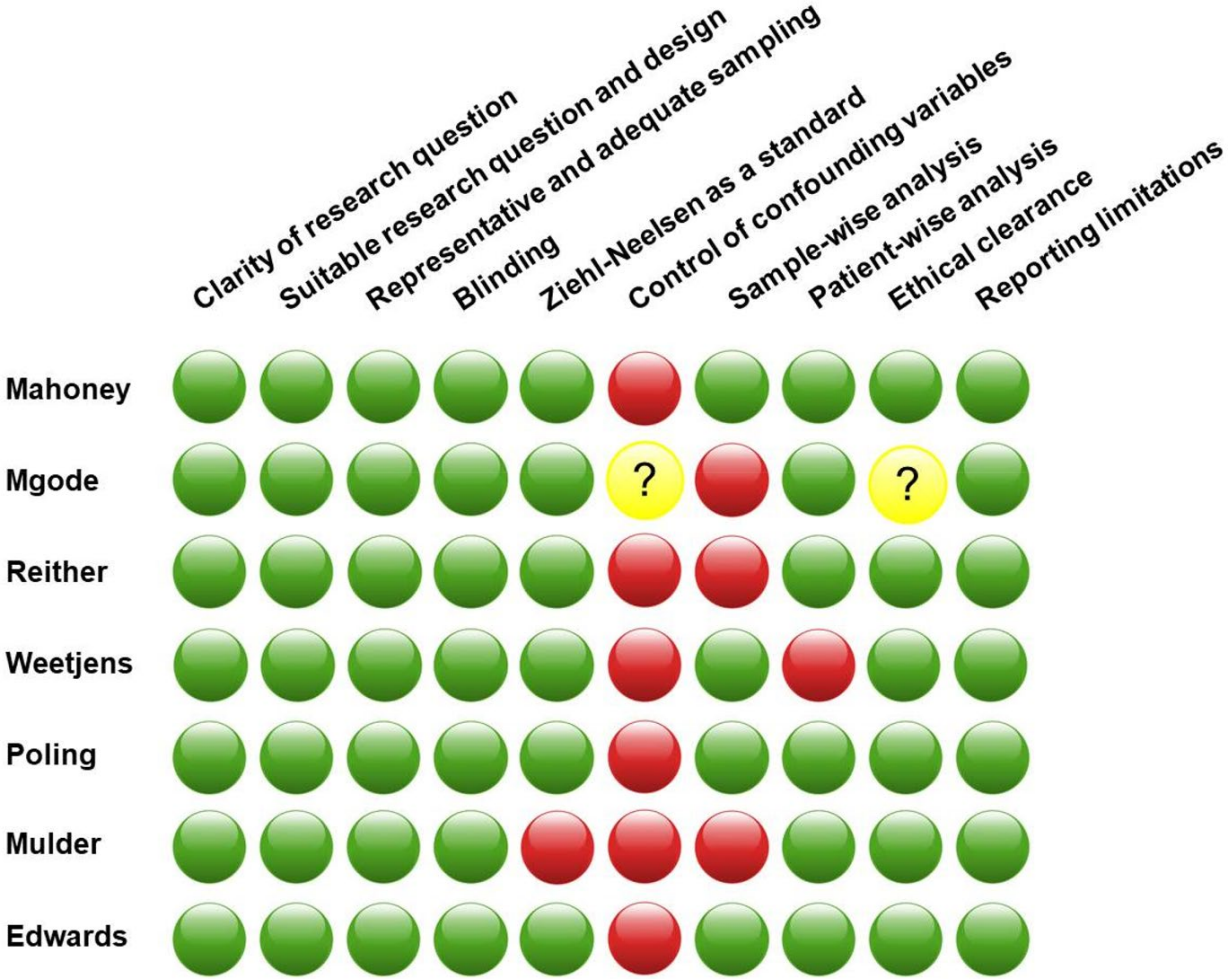
The quality of each study was assessed by answering questions concerning the clarity of research, blindedness, representative and adequate samples, control of confounding variables, research design suitable to answer the research question, ethical clearance, reporting overall sensitivity percentage, reporting overall specificity percentage, and reporting limitations. The presence of non-mycobacterial species in addition to failure to report HIV status were deemed confounding factors. Green = yes; yellow = unclear; red = no.

After examining the seven studies for possible risk of publication bias by the criteria listed in Fig. 6, (with particular emphasis on clarity of the research question and experimental design), we concluded that they are all high-quality studies. All studies were properly blinded. Moreover, all studies reported sensitivity and specificity relative to culturing, ZN, LED FM, or Xpert MTB/RIF assays. The studies also reported limitations and obtained ethical clearance. On the other hand, HIV status of subjects was not distinguishable in any of the included studies. Furthermore, rats' discrimination ability between sputum containing *Mycobacterium* species versus those containing non-mycobacterial species of the respiratory tract was determined only in one study, Mgode et al.^22^. We combined these two facts under the category “failure to control the confounding factors.” Nonetheless, this meta-analysis shows that trained giant African pouched rats can use scent to discriminate sputum of TB individuals from that of healthy subjects at a high accuracy. Deeks’ Funnel Plot Asymmetry test resulted pvalue = 0.09 and pvalue = 0.97, for patient-wise and sample-wise data respectively. The test indicated significant publication bias in patient-wise studies, but no publication bias was detected in sample-wise studies (Supplementary Figures S1 and S2).

## Discussion

Africa has the highest TB-burden in the world, 30% of TB patients have HIV compared to 1.3% in the Eastern Mediterranean region. Additionally, Africa bears 75% of just over one million TB/HIV co-infection incidences that arise each year globally^30^. Therefore, a diagnostic test that is simple, economical, fast, and able to generate results at the point-of-care is needed to test large segments of the population. Rapid diagnosis results in enhanced adherence to and effectiveness of treatment, avoids long-term complications, and reduces the incidence of the disease transmission^26^. DOTS centres in Tanzania and Mozambique trained African giant pouched rats to distinguish sputum samples between TB patients and healthy individuals^28^. Rats in the laboratory were capable of screening 140 sputum samples in 40 min^21^, providing one of the fastest methods available at the cost of approximately 1 USD per sample compared with 1.5 USD, 12–17 USD and 20 USD for smear microscopy, culturing and Xpert, respectively^29^.

A comparison of rat-positive and rat-negative sputa with *M. tuberculosis* versus non-mycobacterial species sputa revealed that sputa detected from *M. catarrhalis*, *S. pneumoniae*, *Candida* sp., *Enterococcus* sp., *Staphylococcus succinus*, and another *Staphylococcus* sp. are significantly different from that of *M. tuberculosis*.

The statistically significant difference in the distribution of rat-positive and rat-negative sputa with *M. tuberculosis* and non-tuberculous species shows that trained rats did not make false-detections with these microorganisms^22^.

Historically, the rat’s olfactory system has been considered primitive. Until recently, cognitive neuroscientists have disregarded odour-guided behaviour^31^. Now we know that the olfactory system has projections to the prefrontal cortex, entorhinal cortex and hippocampus in the brain^31^. These connections carry the acquisition of simple and higher-order instrumental jobs, as well as a memory. It seems that animals with an enhanced perceptive sense of smell are equipped to “think with their noses”^31^. A study seeking to understand the molecular basis for prey identifying its predator found that olfactory-derived defensive mechanism in the prey (like rodents) gave them a strong evolutionary advantage for survival. For example, an examination of 38 mammalian species by quantitative HPLC analysis indicated that many carnivores produced > 3000-fold more 2-phenylethylamine than herbivores. Thus, rodents avoid a 2-phenylethylamine odour source in their natural habitat^32^.

VOCs of active pulmonary tuberculosis derived from the infectious organism may contain biomarkers for the disease^33^. For example, methyl phenylacetate, methyl p-anisate, methyl nicotinate, and o-phenylanisole were predominant in *M. tuberculosis* and *Mycobacterium bovis* cultures grown in vitro^34^. Other cultured *M. tuberculosis*-specific volatiles include 1-methyl-naphthalene, 3-heptanone, methyl-cyclododecane, 2,2,4,6,6-pentamethyl-heptane, 1-methyl-4-(1-methylethyl)-benzene, and 1,4-dimethyl-cyclohexane. These distinctive volatile markers may be the basis for odour detection by rats^35^. Among the VOCs released by *M. tuberculosis* is 2-phenylethanol (PEA) the biosynthetic route of which is via 2-phenylethylamine pathway^36^. The headspace of cultures of *M. bovis* and *Mycobacterium smegmatis* grown on Lowenstein-Jensen supplemented with glycerol were examined by ultra-rapid gas chromatograph with a surface acoustic wave sensor (zNose) and revealed the presence of 2-phenylethanol^37^. We hypothesise that the giant African pouched rats recognise 2-phenylethylamine produced by *M. tuberculosis* along with other disease-specific associated VOC markers. This hypothesis highlights the need for additional studies to identify the exact volatile compounds detected by rats.

One of the limitations is the considerable heterogeneity for both sample-wise and patient-wise experimental set-ups. The other limitation is that all studies examined were carried out by the same group of scientists belonging to APOPO. While this does not necessarily compromise the quality of the conducted research, it does highlight the importance of having other research teams test the rat-sniffing method, especially in high TB/HIV prevalence areas. In additional TB detection studies, it would be advantageous to publish separate statistical analyses of the performance of rats on non-tuberculosis samples and HIV status. The other shortcoming of the rat-sniffing method is that it does not meet current WHO-recommended standards and is not a replacement for smear microscopy or Xpert MTB/RIF methods. Instead, the rat-sniffing method could serve as a screen for identifying probable TB patients in high-throughput circumstances where the use of other technologies would be too expensive. From the point of view of global acceptance of TB detection performed by rats, it would be necessary to broaden the geographical area tested.

At APOPO, rat-positive samples were re-evaluated using the standard ZN method for confirmation; results show that the accuracy of using rats is as good as using ZN as the primary method for detecting TB. We frequently calculated sensitivity and specificity values of rats over 100% for TB when considering only the first ZN test, without further ZN confirmation. The “over 100%” needs to be explained. Normally it is impossible, but there can be a strange situation, when the reference method has lower accuracy. What are the real positive and real negative? Let us describe the sequence of detection in the analysed publications. Initially, TB patients were determined by ZN positivity. Then rats identified some new positive samples or patients in the ZN control group. These rat positives were re-evaluated by ZN. The final evaluation regarded every ZN-positive test as patients or patient samples; even if they were identified in the second-round ZN evaluation. The unusual “over 100%” was calculated for the rats, when the first ZN was regarded the reference but real positives and real negatives for the rats were calculated considering the second ZN detection. While these results cannot be interpreted, they do show that the accuracy of rats may be better than a single ZN evaluation for identifying TB.

Using giant African pouched rats for large-scale screening and diagnosis is advantageous for countries with low economic status and high incidences of tuberculosis due to it being a relatively cheap and rapid method. Nota bene, a rat can identify a hundreds of samples in less than 20 min. While this task lasts 4 days for a trained microscopist technician^38^. Our systematic review and meta-analysis validated the African pouched rat-sniffing method as a first-line screening tool for TB.

## Supplementary Information

Supplementary Information.
