# Supplementary material for: Rats sniff out pulmonary tuberculosis from sputum: a diagnostic accuracy meta-analysis
Source: Sci Rep. 2021 Jan 21;11:1877. doi: 10.1038/s41598-021-81086-x (PMC7820466; doi:10.1038/s41598-021-81086-x)
Supplement: Supplementary file 1 — Supplementary Information. [file 41598_2021_81086_MOESM1_ESM.doc]

Supplementary Information

**Rats sniff out pulmonary tuberculosis from sputum: a diagnostic accuracy meta-analysis**

Reem Kanaan, Nelli Farkas, Péter Hegyi, Alexandra Soós, Dávid Hegyi, Katalin Németh, Orsolya Horváth, Judit Tenk, Alexandra Mikó, Andrea Szentesi, Márta Balaskó, Zsolt Szakács, Andrea Vasas, Dezső Csupor, Zoltán Gyöngyi

Supplementary Table S1


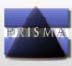
**PRISMA 2009 Checklist**

| **Section/topic** | **#** | **Checklist item** | **Reported on page #** |
| --- | --- | --- | --- |
| **TITLE** | | |  |
| Title | 1 | Identify the report as a systematic review, meta-analysis, or both. | 1 |
| **ABSTRACT** | | |  |
| Structured summary | 2 | Provide a structured summary including, as applicable: background; objectives; data sources; study eligibility criteria, participants, and interventions; study appraisal and synthesis methods; results; limitations; conclusions and implications of key findings; systematic review registration number. | 2 |
| **INTRODUCTION** | | |  |
| Rationale | 3 | Describe the rationale for the review in the context of what is already known. | 3-4 |
| Objectives | 4 | Provide an explicit statement of questions being addressed with reference to participants, interventions, comparisons, outcomes, and study design (PICOS). | 4 |
| **METHODS** | | |  |
| Protocol and registration | 5 | Indicate if a review protocol exists, if and where it can be accessed (e.g., Web address), and, if available, provide registration information including registration number. | 4 |
| Eligibility criteria | 6 | Specify study characteristics (e.g., PICOS, length of follow-up) and report characteristics (e.g., years considered, language, publication status) used as criteria for eligibility, giving rationale. | 4 |
| Information sources | 7 | Describe all information sources (e.g., databases with dates of coverage, contact with study authors to identify additional studies) in the search and date last searched. | 4 |
| Search | 8 | Present full electronic search strategy for at least one database, including any limits used, such that it could be repeated. | 4 |
| Study selection | 9 | State the process for selecting studies (i.e., screening, eligibility, included in systematic review, and, if applicable, included in the meta-analysis). | 4 |
| Data collection process | 10 | Describe method of data extraction from reports (e.g., piloted forms, independently, in duplicate) and any processes for obtaining and confirming data from investigators. | 5 |
| Data items | 11 | List and define all variables for which data were sought (e.g., PICOS, funding sources) and any assumptions and simplifications made. | 5, 18 |
| Risk of bias in individual studies | 12 | Describe methods used for assessing risk of bias of individual studies (including specification of whether this was done at the study or outcome level), and how this information is to be used in any data synthesis. | 5 |
| Summary measures | 13 | State the principal summary measures (e.g., risk ratio, difference in means). | 5 |
| Synthesis of results | 14 | Describe the methods of handling data and combining results of studies, if done, including measures of consistency (e.g., I^2^) for each meta-analysis. | 5 |

Page 1 of 2

| **Section/topic** | **#** | **Checklist item** | **Reported on page #** |
| --- | --- | --- | --- |
| Risk of bias across studies | 15 | Specify any assessment of risk of bias that may affect the cumulative evidence (e.g., publication bias, selective reporting within studies). | 5 |
| Additional analyses | 16 | Describe methods of additional analyses (e.g., sensitivity or subgroup analyses, meta-regression), if done, indicating which were pre-specified. | - |
| **RESULTS** | | |  |
| Study selection | 17 | Give numbers of studies screened, assessed for eligibility, and included in the review, with reasons for exclusions at each stage, ideally with a flow diagram. | 6, Fig. 1 |
| Study characteristics | 18 | For each study, present characteristics for which data were extracted (e.g., study size, PICOS, follow-up period) and provide the citations. | 6, Table 1 |
| Risk of bias within studies | 19 | Present data on risk of bias of each study and, if available, any outcome level assessment (see item 12). | 6, |
| Results of individual studies | 20 | For all outcomes considered (benefits or harms), present, for each study: (a) simple summary data for each intervention group (b) effect estimates and confidence intervals, ideally with a forest plot. | 7, Fig. 2 and Fig. 3 |
| Synthesis of results | 21 | Present results of each meta-analysis done, including confidence intervals and measures of consistency. | 7 |
| Risk of bias across studies | 22 | Present results of any assessment of risk of bias across studies (see Item 15). | 7, Fig. 4 |
| Additional analysis | 23 | Give results of additional analyses, if done (e.g., sensitivity or subgroup analyses, meta-regression [see Item 16]). | - |
| **DISCUSSION** | | |  |
| Summary of evidence | 24 | Summarize the main findings including the strength of evidence for each main outcome; consider their relevance to key groups (e.g., healthcare providers, users, and policy makers). | 8 |
| Limitations | 25 | Discuss limitations at study and outcome level (e.g., risk of bias), and at review-level (e.g., incomplete retrieval of identified research, reporting bias). | 9 |
| Conclusions | 26 | Provide a general interpretation of the results in the context of other evidence, and implications for future research. | 10 |
| **FUNDING** | | |  |
| Funding | 27 | Describe sources of funding for the systematic review and other support (e.g., supply of data); role of funders for the systematic review. | 14 |

PRISMA= Preferred Reporting Items for Systematic Reviews and Meta-Analyses

*From:*  Moher D, Liberati A, Tetzlaff J, Altman DG, The PRISMA Group (2009). Preferred Reporting Items for Systematic Reviews and Meta-Analyses: The PRISMA Statement. PLoS Med 6(7): e1000097. doi:10.1371/journal.pmed1000097

For more information, visit: [**www.prisma-statement.org**](http://www.prisma-statement.org/).

Supplementary Table S2. 2 by 2 tables of extracted data.

| Reference method | Publication | sample wise | |  |  |  |  | patient wise | |  |  |  |
| --- | --- | --- | --- | --- | --- | --- | --- | --- | --- | --- | --- | --- |
| ZN | Mahoney et al. 2010 |  |  |  |  |  |  |  |  |  |  |  |
|  |  |  |  |  |  |  |  |  |  |  |  |  |
|  |  |  |  | + | - |  |  |  |  | + | - |  |
|  |  | rat | + | 3389 | 5421 | 8810 |  | rat | + | 2290 | 3128 | 5418 |
|  |  |  | - | 418 | 17437 | 17855 |  |  | - | 97 | 6814 | 6911 |
|  |  |  |  |  |  |  |  |  |  |  |  |  |
|  |  |  |  | 3807 | 22858 | 26665 |  |  |  | 2387 | 9942 | 12329 |
|  |  |  |  |  |  |  |  |  |  |  |  |  |
|  | Reither etal. 2015 |  |  |  |  |  |  |  |  | + | - |  |
|  |  |  |  |  |  |  |  | rat | + | 52 | 37 | 89 |
|  |  |  |  |  |  |  |  |  | - | 28 | 129 | 157 |
|  |  |  |  |  |  |  |  |  |  | 80 | 166 | 246 |
|  |  |  |  |  |  |  |  |  |  |  |  |  |
|  | Weetjens et al. 2009 |  |  | + | - |  |  |  |  |  |  |  |
|  |  | rat | + | 303 | 95 | 398 |  |  |  |  |  |  |
|  |  |  | - | 42 | 2157 | 2199 |  |  |  |  |  |  |
|  |  |  |  | 345 | 2252 | 2597 |  |  |  |  |  |  |
|  |  |  |  |  |  |  |  |  |  |  |  |  |
|  | Poling et al. 2010 |  |  | + | - |  |  |  |  | + | - |  |
|  |  | rat | + | 3201 | 2085 | 5286 |  | rat | + | 1955 | 798 | 2753 |
|  |  |  | - | 213 | 17602 | 17815 |  |  | - | 68 | 7702 | 7770 |
|  |  |  | Total | 3414 | 19687 | 23101 |  |  | Total | 2023 | 8500 | 10523 |
|  |  |  |  |  |  |  |  |  |  |  |  |  |
|  | Edwards et al. 2016 |  |  | + | - |  |  |  |  |  |  |  |
|  |  | rat | + | 61 | 164 | 225 |  |  |  |  |  |  |
|  |  |  | - | 15 | 578 | 593 |  |  |  |  |  |  |
|  |  |  | Total | 76 | 742 | 818 |  |  |  |  |  |  |
|  |  |  |  |  |  |  |  |  |  |  |  |  |
| culture | Reither et al. 2015 |  |  |  |  |  |  |  |  | + | - |  |
|  |  |  |  |  |  |  |  | rat | + | 62 | 27 | 89 |
|  |  |  |  |  |  |  |  |  | - | 47 | 110 | 157 |
|  |  |  |  |  |  |  |  |  |  | 109 | 137 | 246 |
|  |  |  |  |  |  |  |  |  |  |  |  |  |
|  | Weetjens et al. 2009 |  |  | + | - |  |  |  |  |  |  |  |
|  |  | rat | + | 303 | 95 | 398 |  |  |  |  |  |  |
|  |  |  | - | 42 | 2157 | 2199 |  |  |  |  |  |  |
|  |  |  |  | 345 | 2252 | 2597 |  |  |  |  |  |  |
|  |  |  |  |  |  |  |  |  |  |  |  |  |
|  | Mulder et al. 2017 |  |  |  |  |  |  |  |  | + | - |  |
|  |  |  |  |  |  |  |  | rat | + | 219 | 180 | 399 |
|  |  |  |  |  |  |  |  |  | - | 126 | 246 | 372 |
|  |  |  |  |  |  |  |  |  | Total | 345 | 426 | 771 |
|  |  |  |  |  |  |  |  |  |  |  |  |  |
| ZN + | Mgode et al. 2012 |  |  |  |  |  |  |  |  | + | - |  |
| culture |  |  |  |  |  |  |  | rat | + | 45 | 63 | 108 |
|  |  |  |  |  |  |  |  |  | - | 11 | 165 | 176 |
|  |  |  |  |  |  |  |  |  |  | 56 | 228 | 284 |
|  |  |  |  |  |  |  |  |  |  |  |  |  |
| LED FM | Edwards et al. 2016 |  |  | + | - |  |  |  |  |  |  |  |
|  |  | rat | + | 86 | 139 | 225 |  |  |  |  |  |  |
|  |  |  | - | 36 | 557 | 593 |  |  |  |  |  |  |
|  |  |  | Total | 122 | 696 | 818 |  |  |  |  |  |  |
|  |  |  |  |  |  |  |  |  |  |  |  |  |
| Xpert | Mulder et al. 2017 |  |  |  |  |  |  |  |  | + | - |  |
|  |  |  |  |  |  |  |  | rat | + | 153 | 242 | 395 |
|  |  |  |  |  |  |  |  |  | - | 72 | 298 | 370 |
|  |  |  |  |  |  |  |  |  | Total | 225 | 540 | 765 |

Raw data from the analysed publications. The rows in the separate tables show the number of samples or cases that were judged positive (rat +) or negative (rat -) by rats (*Cricetomys gambianus*). The columns in the separate tables indicate the number positive (+) or negative (-) samples or cases verified by ZN, LED FM or Xpert methods. Sample wise = identification of tuberculosis (TB) positivity or negativity of sputum samples, Patient wise = identification of individuals with or without TB.

ZN = Ziehl-Neelsen stain-based, direct microscopy; LED FM = Light-emitting diode fluorescence microscopy; Xpert = MTB/RIF test that detects *Mycobacterium tuberculosis* complex (MTBC) and resistance to rifampicin (RIF) on the GeneXpert multi-disease platform.

Supplementary Table S3. True positive (tp), false positive (fp), false negative (fn) and true negative (tn) values of analysed studies.

| Studies | subgroups | tp | fp | fn | tn |
| --- | --- | --- | --- | --- | --- |
| Mahoney et al., 2010 | patient wise | 2290 | 3128 | 97 | 6814 |
| Reither et al., 2015 | patient wise | 52 | 37 | 28 | 129 |
| Poling et al., 2010 | patient wise | 1955 | 798 | 68 | 7702 |
| Mgode et al., 2012 | patient wise | 45 | 63 | 11 | 165 |
| Reither et al., 2015 (culture) | patient wise | 62 | 27 | 47 | 110 |
| Mulder et al., 2017 | patient wise | 219 | 180 | 126 | 246 |
| Mulder et al., 2017 (Xpert) | patient wise | 153 | 242 | 72 | 298 |
| Mahoney et al., 2010 | sample wise | 3389 | 5421 | 418 | 17437 |
| Weetjens et al., 2009 | sample wise | 303 | 95 | 42 | 2157 |
| Poling et al., 2010 | sample wise | 3201 | 2085 | 213 | 17602 |
| Edwards et al., 2016 | sample wise | 61 | 164 | 15 | 578 |
| Weetjens et al., 2009 (culture) | sample wise | 303 | 95 | 42 | 2157 |
| Edwards et al., 2016 (LED FM) | sample wise | 86 | 139 | 36 | 557 |

The reference method was ZN, unless other method is indicated in brackets. ZN = Ziehl-Neelsen stain-based, direct microscopy; LED FM = Light-emitting diode fluorescence microscopy; Xpert = MTB/RIF test that detects *Mycobacterium tuberculosis* complex (MTBC) and resistance to rifampicin (RIF) on the GeneXpert multi-disease platform.

Supplementary Figure S1. Publication bias analysis for patient-wise datasets.


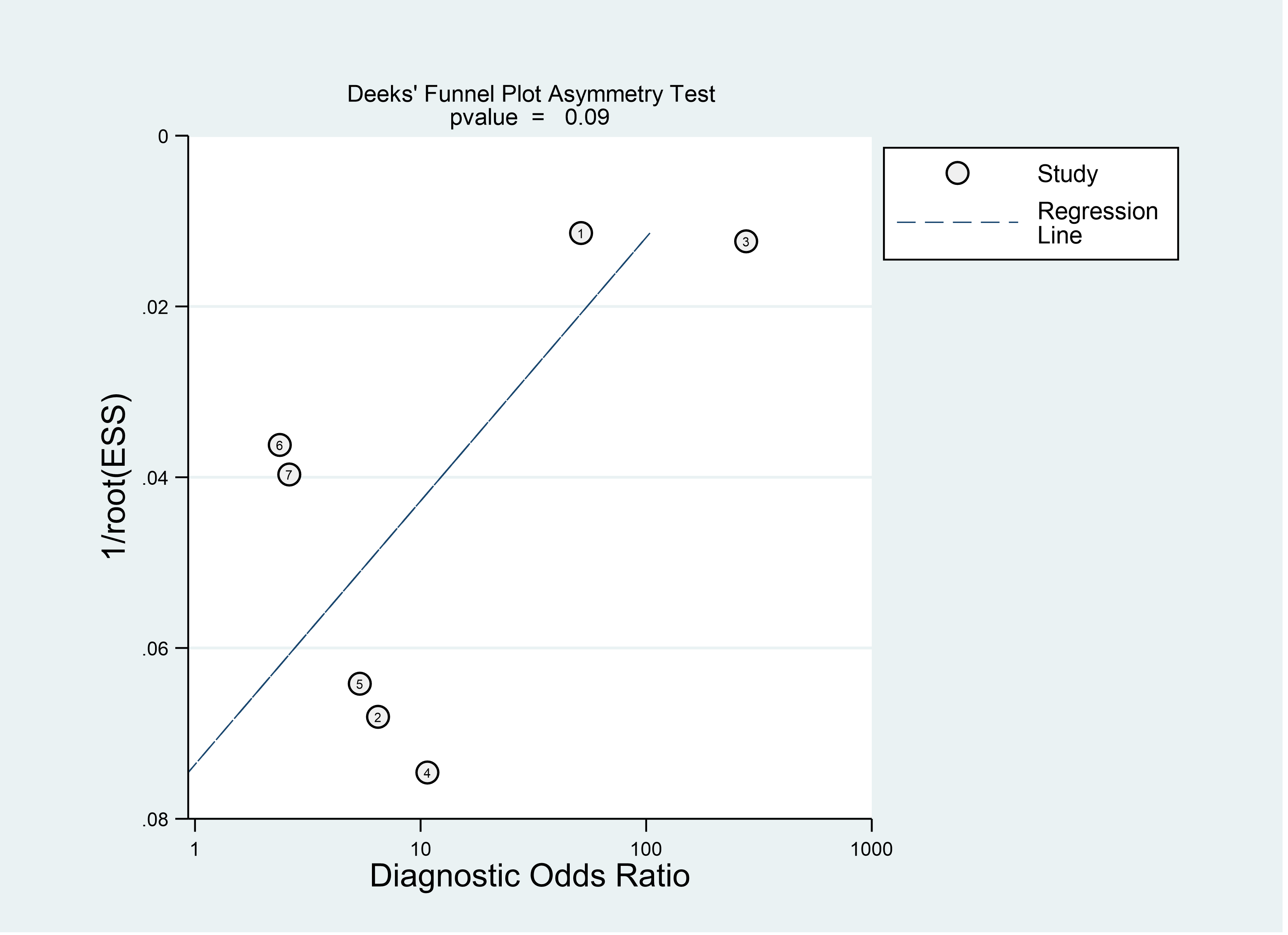


Publications: 1. Mahoney et al., 2010; 2. Reither et al., 2015; 3. Poling et al., 2010; 4. Mgode et al., 2012; 5. Reither et al., 2015 (culture); 6. Mulder et al., 2017; 7. Mulder et al., 2017 (Xpert). The reference method was ZN, unless other method is indicated in brackets. Publication bias was detected (Deeks’ Funnel Plot Asymmetry test pvalue=0.09). ZN = Ziehl-Neelsen stain-based, direct microscopy; LED FM = Light-emitting diode fluorescence microscopy; Xpert = MTB/RIF test that detects *Mycobacterium tuberculosis* complex (MTBC) and resistance to rifampicin (RIF) on the GeneXpert multi-disease platform; effective sample size = ESS.

Supplementary Figure S2. Publication bias analysis for sample-wise datasets.


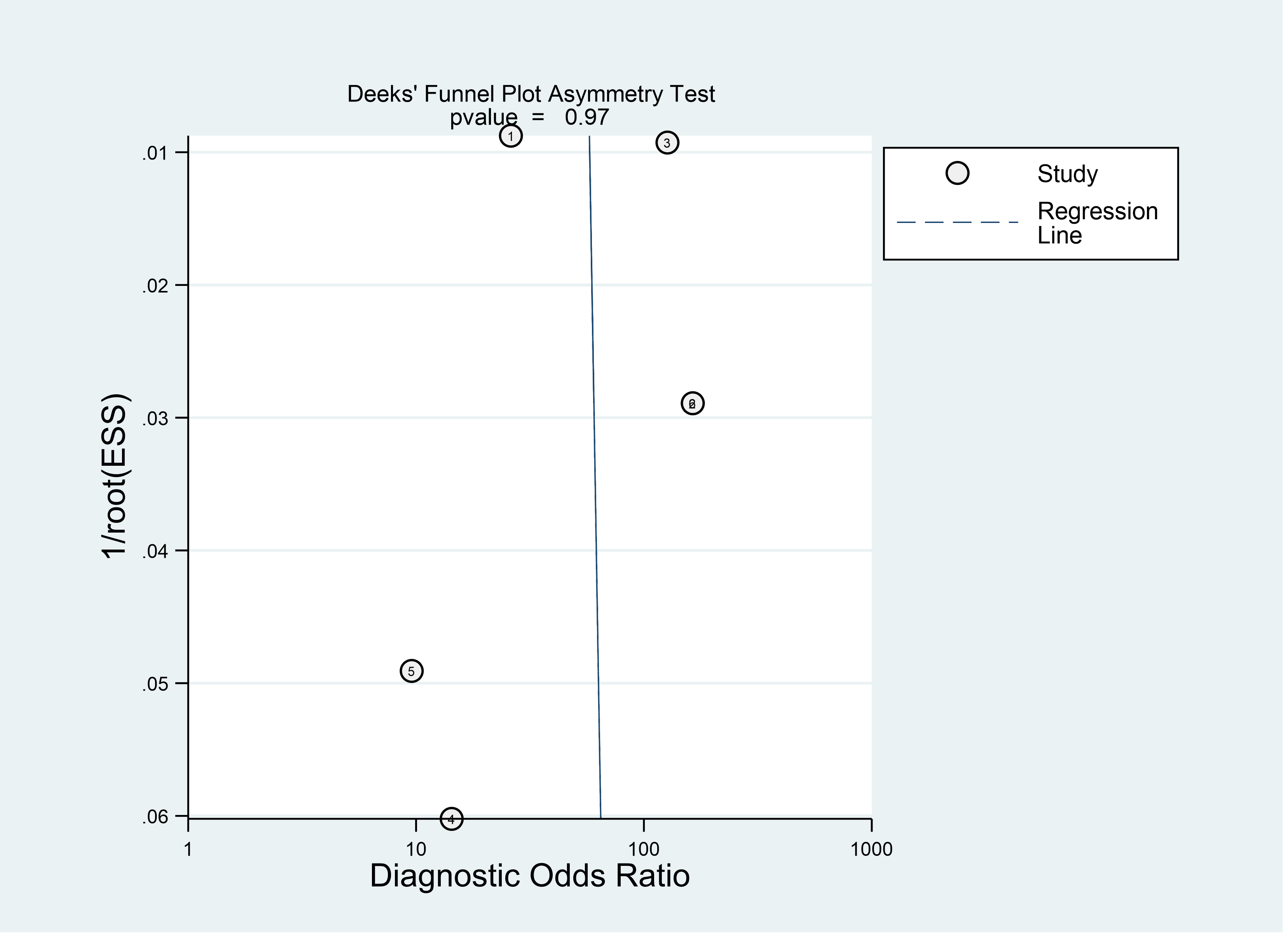


Publications: 1. Edwards et al., 2016; 2. Edwards et al., 2016 (LED FM); 3. Mahoney et al., 2010; 4. Poling et al., 2010; 5. Weetjens et al., 2009; 6. Weetjens et al., 2009 (culture). The reference method was ZN, unless other method is indicated in brackets. There is no publication bias detected (Deeks’ Funnel Plot Asymmetry test pvalue=0.97). ZN = Ziehl-Neelsen stain-based, direct microscopy; LED FM = Light-emitting diode fluorescence microscopy; Xpert = MTB/RIF test that detects *Mycobacterium tuberculosis* complex (MTBC) and resistance to rifampicin (RIF) on the GeneXpert multi-disease platform; effective sample size = ESS.
